# A cross-cultural comparison of health-related quality of life and its associated factors among older women in Vietnam and Australia

**DOI:** 10.1186/s13104-018-3282-0

**Published:** 2018-03-13

**Authors:** Tiet-Hanh Dao-Tran, Charrlotte Seib, Lee Jones, Debra Anderson

**Affiliations:** 1 Centre for Work, Organisation, and Wellbeing, Griffith University, Brisbane, Australia; 2 University of Medicine and Pharmacy at Ho Chi Minh City, Ho Chi Minh City, Vietnam; 3 School of Nursing and Midwifery, Griffith University, Gold Coast, Australia; 4 Institute of Health and Biomedical Innovation, Queensland University of Technology, Brisbane, Australia; 5 School of Nursing and Midwifery, Griffith University, Gold Coast, Australia

**Keywords:** Health-related quality of life, Lifestyles, Chronic diseases, Older women, Cross-cultural comparison

## Abstract

**Objective:**

This study compared health-related quality of life and its associated factors among 305 women in Vietnam and 175 women in Australia aged 60–71. Descriptive analyses, Chi square, independent t-tests, and General Linear Models were used for data analysis.

**Results:**

After controlling for socio-demographics, lifestyles, and chronic diseases, older women in Vietnam had lower levels of physical health but similar levels of mental health to those in Australia. In both populations, chronic disease and diet were associated with physical health; physical activity was related to mental health. In Australia, physical activity, exercise, and Body Mass Index were also associated with physical health; age, alcohol consumption, and sleep were also linked with mental health. In Vietnam, age and marital status were also related to physical health; chronic diseases and diet were also correlated with mental health. These findings suggested that interventions developed in Australia targeting the management of diet and physical activity, may be useful for older women in Vietnam. However, future interventions in Vietnam need to be tailored to account for different age groups, marital status, and the number of chronic diseases experienced. Further investigation into the contributions of cultural factors to health-related quality of life is recommended.

## Introduction

Older women usually reported low levels of health related quality of life (HRQOL) [1–4], raising concern about HRQOL from gender and ageing perspectives. In Vietnam, the number of people aged 60 and over is expected to increase to 20% of the total population by 2050 [5]. Among this older population, women comprise a higher proportion (58.4%) [5]. In addition, older women in Vietnam have been subject to various life stressors, which has influenced their lifestyles, health and wellbeing [6]. However, knowledge about HRQOL among older women in Vietnam is still limited.

Comparisons of HRQOL and its associated factors across cultures revealed variations among people from different backgrounds [7–9]. These comparisons were conducted among people living in similar populations—for example: among those living in developed countries together [7, 8], among those living in developing countries together [9]—or between people with different cultural background but living in the same context [10]. Similarities and differences in HRQOL and its associated factors between those living in developed countries compared to those living in developing countries—in particular between Vietnam and Australia—is still unclear.

A cross-cultural comparison between Vietnam and Australia might further provide useful directions to help utilize and optimize the resources, which were developed to promote HRQOL in Australia [11, 12] for those in Vietnam and this study was conducted to address the following research questions:

1. Are there similarities in HRQOL and its associated factors among older women in Vietnam and Australia?
2. What are the differences in HRQOL and its associated factors among older women in Vietnam and Australia?

## Main text

### Methods

This cross-sectional study used secondary data from Vietnam and Australia. In Vietnam, data were originally collected in 2013 from 440 female citizens aged 60+ living in urban and rural suburbs in the South of Vietnam; the selection was stratified random [13, 14]. In Australia, data was collected in 2012 from 179 female citizens aged 55–71 living in urban and rural suburbs in South East Queensland. The sample was previously selected at random for participation in longitudinal research conducted from 2001 to 2013 [7, 15, 16]. To achieve a consistent age group among the participants, only data from women aged 60–71 were used for this comparison, including 305 older women in Vietnam and 175 older women in Australia. This sample size is sufficient for the study power statistical analysis.

Data from Vietnam and Australia were measured using the same scales including General Sleep Disturbance Scale [17], and the Short-Form 12 (SF-12) [18]). Self-report questionnaires were administered during an interview during data collection in Vietnam and; self-reported questionnaires were applied for the Australian data collection. Details of the populations, samples and sampling methods, and measurements have been published elsewhere [6, 7, 13–16, 19, 20].

Statistical Package for Social Sciences (SPSS) 23.0 was used for data analysis [21]. Descriptive analyses, Chi square tests and independent t-tests were used to describe the samples’ characteristics. General Linear Models (GLM) with purposeful selection approach [22] were also used to compare HRQOL and factors associated with HRQOL among older women in Vietnam and Australia. First, the interactions between factors and nation of residency were examined. Following, bivariate associations were performed to select the associations having p < .25 for an initial multivariable model. All insignificant associations in the initial model were excluded. Next, each excluded variable took turns to come back to the model to check if they were significant and/or were confounders (if they changed the existing model predictor coefficients by 20% or more). Finally, the interactions among factors in the final model were examined in order to finalize the model. The significance level was set at .05 [23].

### Results

As 45.7% of participants in Australia did not disclose their income, income were excluded from the study analysis. As seen in Table 1, physical activity were similar between women in Vietnam and Australia (p > .05). However, participants in Vietnam were slightly younger (64.72 (SD = 3.5) vs. 65.88 (SD = 2.9)), less likely to live with their partners (58% vs. 76%), and be unemployed than Australian participants (34.1% vs. 22.9%). Majority of participants in Vietnam had low education levels (72.1% with primary education or less), while more than one-third of participants in Australia had higher degrees (33.9% with diploma and university levels).

**Table 1:** Sociodemographic characteristics, lifestyles and chronic diseases among older women in Vietnam (n = 305) and Australia (n = 175)

	Vietnam, n (%)	Australia, n (%)	Differences
Age (mean (SD))	64.72 (3.5)	65.88 (2.9)	t(478) = 14.09*
Married/de facto	177 (58)	130 (76)	χ^2^ (1) = 15.49*
Highest education			
Primary/less	220 (72.1)	31 (18.1)	χ^2^ (3) = 144.14*
Junior school	44 (14.4)	60 (35.1)	
Senior school	26 (8.5)	22 (12.9)	
Diploma and university	15 (4.9)	58 (33.9)	
Employed	104 (34.1)	40 (22.9)	χ^2^ (1) = 6.69*
Having ≥ 5 servings of vegetable daily	132 (43.3)	129 (73.7)	χ^2^ (1) = 41.52*
Having ≥ 2 servings of fruit daily	128 (42.0)	138 (78.9)	χ^2^ (1) = 61.25*
Alcohol consumption	31 (10.2)	146 (83.4)	χ^2^ (1) = 256.42*
Tobacco smoking	5 (1.6)	59 (33.7)	χ^2^ (1) = 99.00*
Physical activity			χ^2^ (3) = 13.90
Very active	55 (18.0)	15 (8.8)	
Moderately active	172 (56.4)	115 (67.3)	
Mildly active	56 (18.4)	37 (21.6)	
Sedentary	22 (7.2)	4 (2.3)	
Exercise status			χ^2^ (2) = 119.15*
None	189 (62.0)	39 (22.8)	
Not daily	17 (5.6)	112 (65.5)	
Daily	99 (32.5)	20 (11.7)	
Sleep disturbance	116 (38.0)	14 (9.1)	χ^2^ (1) = 42.22*
BMI			χ^2^ (3) = 110.51*
Underweight	17 (5.6)	1 (.6)	
Normal	202 (66.2)	56 (32.7)	
Overweight	70 (23.0)	42 (24.6)	
Obese	16 (5.2)	72 (42.1)	
Chronic diseases	1.61 (.07)	1.17 (.07)	t(478) = − 3.99*
Hypertension	153 (50.2)	58 (33.9)	χ^2^ (1) = 11.73*
Heart disease	80 (26.2)	2 (1.2)	χ^2^ (1) = 28.26*
Stroke	14 (4.6)	2 (1.2)	χ^2^ (1) = 3.95**
Type 2 diabetes	36 (11.8)	15 (8.8)	χ^2^ (1) = 1.05
Breast cancer	2 (.7)	6 (3.5)	χ^2^ (1) = 5.40**
Arthritis	155 (50.8)	73 (42.7)	χ^2^ (1) = 2.90
Osteoporosis	52 (17.0)	33 (19.3)	χ^2^ (1) = .38
Depression	1 (.3)	26 (15.2)	χ^2^ (1) = 45.32*

* p < .05; ** p  < .01;

Participants in Vietnam were less likely to have a healthy diet (43.3% vs. 73.7% having ≥ 5 servings of vegetable daily, 42% vs. 78.9% having ≥ 2 servings of fruit daily), consume alcohol (10.2%vs. 83.4%) and tobacco (1.6% vs. 33.7%), practise exercise (22.8% vs. 62.0%), and be overweight or obese (28.2% vs. 66.7%), but had a prevalence of sleep disturbance four times higher (38.0% vs. 9.1%), and more chronic conditions (1.61 (SD = .07) vs. 1.17 (SD = .07) than their Australian counterparts. The prevalence of type 2 diabetes, arthritis, and osteoporosis were similar between the participants in Vietnam and Australia (p > .05). The prevalence of hypertension (50.2% vs. 33.9%), heart disease (26.2% vs. 1.2%), and stroke (4.6% vs. 1.2%) was significantly higher among the participants in Vietnam. The prevalence of breast cancer (3.5% vs. .7%) and depression (15.2% vs. .3%) was significantly higher among the participants in Australia.

Among participants in Vietnam, crude physical health mean scores were 37.7 (SD = 13.2), and crude mental health mean scores were 54.3 (SD = 10.9). Among participants in Australia, crude physical health mean scores were 46.4 (SD = 10.0), and crude mental health mean scores were 55.1 (SD = 7.4).

As seen in Table 1, participants in Vietnam and Australia had a number of differences in sociodemographic characteristics, lifestyles, and the number of chronic diseases, which may confound the comparison of HRQOL using crude values. Thus, GLM was required to adjust the comparison and GLM (Table 2) demonstrates no interactions between sociodemographic characteristics, lifestyles, and the number of chronic diseases and nation of residency (p > .05). After controlling for personal characteristics, lifestyles, and the number of chronic diseases, Australian participants still had higher levels of adjusted physical health than those in Vietnam (OR = 6.8, p < .01), but similar levels of adjusted mental health (p > .05).

**Table 2:** Factors associated HRQOL among older women in Vietnam and Australia (n = 480)

Parameters	β	Sig	95% CI	
Physical health				
Correct model		< .01		
Intercept	67.13	< .01	46.96	87.29
Nation of residency				
Australians	6.80	< .01	3.55	10.04
Vietnamese	Ref		Ref	Ref
Age	− .33	< .05	− .64	− .02
Number of chronic diseases	− 2.96	< .01	− 3.88	− 2.03
Fruit consumption				
≥ 2 servings daily	4.81	< .01	2.56	7.06
< 2 servings daily	Ref		Ref	Ref
Daily physical activity level				
Very active	− .11	< .01	− 3.09	2.87
Sedentary	− 5.60		− 10.17	− 1.04
Mildly active	− 4.35		− 7.03	− 1.66
Moderately active	Ref		Ref	Ref
Exercise status				
None	− 3.08	< .05	− 5.57	− .59
Not daily	− 3.25		− 6.70	.20
Daily	Ref		Ref	Ref
Sleep disturbance				
Yes	− 2.89	< .05	− 5.27	− .51
No	Ref		Ref	Ref
BMI				
Obese	− 4.20	< .05	− 7.39	− 1.00
Underweight	− 6.28		− 11.64	− .92
Overweight	− 1.70		− 4.23	.83
Normal	Ref		Ref	Ref
Adjusted R square				28.3%
Mental health				
Correct model		< .01		
Intercept	56.25	< .01	54.33	58.16
Nation of residency				
Australians	.17	.90	− 2.51	2.86
Vietnamese	Ref		Ref	Ref
Number of chronic diseases	− .88	< .05	− 1.63	− .13
Fruit consumption				
≥ 2 servings daily	3.15	< .01	1.30	5.00
< 2 servings daily	Ref	Ref	Ref	Ref
Daily physical activity level				
Very active	− 5.71	< .01	− 8.19	− 3.22
Sedentary	− 5.72		− 9.60	− 1.85
Mildly active	− .55		− 2.77	1.67
Moderately active	Ref		Ref	Ref
Alcohol consumption				
Yes	− 2.55	.05	− 5.11	.00
No	Ref		Ref	Ref
Adjusted R square				8.2%

In regards to factors associated with HRQOL, the study found that among participants in Vietnam, age, chronic diseases, marital status, and fruit consumption were significantly associated with their physical health (p < .01), explaining the 16.9% variance of their physical health scores. Chronic diseases, fruit consumption, and daily physical activity levels were significantly associated with mental health among participants in Vietnam (p < .01), explaining 10.1% variance of their mental health scores. Among participants in Australia, chronic diseases, vegetable consumption, daily physical activity levels, exercise, and BMI were significantly associated with their physical health scores (p < .01), explaining 32.1% variance of their physical health. Age, alcohol consumption, daily physical activity levels, and sleep disturbance were significantly associated with their mental health, explaining 21.4% variance of their mental health scores. Details about contributions of each factors to physical and mental health are presented in Table 3.

**Table 3:** Factors associated with HRQOL in relation to nation of residency (n = 305 and 175)

Parameters	β	Sig	95% CI	
Physical health/ Vietnam (n = 305)				
Correct model		< .01		
Intercept	72.06	< .01	47.03	97.09
Age	− .46	< .05	− .85	− .07
Number of chronic diseases	− 3.39	< .01	− 4.49	− 2.29
Marital status				
Married/ partnered	− 2.78	< .05	− 5.56	.00
Others	Ref		Ref	Ref
Fruit consumptions				
≥ 2 servings daily	5.09	< .01	2.32	7.86
< 2 servings daily	Ref		Ref	Ref
Adjusted R square				16.9%
Mental health/ Vietnam (n = 305)				
Correct model		< .01		
Intercept	56.86	< .01	54.51	59.22
Number of chronic diseases	− 1.35	< .05	− 2.32	− .37
Fruit consumptions				
≥ 2 servings daily	3.13	< .01	.72	5.54
< 2 servings daily	Ref		Ref	Ref
Daily physical activity level				
Very active	− 7.08	< .01	− 10.25	− 3.92
Sedentary	− 4.45		− 9.20	.30
Mildly active	− .31		− 3.50	2.87
Moderately active	Ref		Ref	Ref
Adjusted R square				10.1%
Physical health/ Australia (n = 175)				
Correct model		< .01		
Intercept	57.17	< .01	51.85	62.49
Number of chronic diseases	− 3.51	< .01	− 4.84	− 2.19
Vegetable consumptions				
≥ 5 servings daily	3.06	< .05	.11	6.01
< 5 servings daily	Ref		Ref	Ref
Daily physical activity level				
Very active	2.82	< .01	− 1.89	7.54
Sedentary	− 7.50		− 15.90	.90
Mildly active	− 4.82		− 8.00	− 1.64
Moderately active	2.82		− 1.89	7.54
Exercise status				
None	− 7.66	< .01	− 12.38	− 2.95
Not daily	− 5.67		− 9.86	− 1.49
Daily	Ref		Ref	Ref
BMI				
Obese	− 4.37	< .05	− 7.43	− 1.30
Underweight	− 3.85		− 20.26	12.57
Overweight	− 2.84		− 6.22	.53
Normal	Ref		Ref	Ref
Adjusted R square				32.1%
Mental health/ Australia (n = 175)				
Correct model				
Intercept	4.00	.74	− 19.97	27.98
Age	.84	< .01	.48	1.21
Alcohol drinking				
Yes	− 4.09	< .01	− 6.94	− 1.23
No	Ref		Ref	Ref
Daily physical activity level				
Very active	− 2.22	< .05	− 5.85	1.42
Sedentary	− 9.64		− 16.09	− 3.19
Mildly active	− .82		− 3.36	1.71
Moderately active	Ref		Ref	Ref
Sleep disturbance				
Yes	− 5.49	< .01	− 9.07	− 1.92
No	Ref		Ref	Ref
Adjusted R square				21.4%

### Discussion and conclusion

Comparing HRQOL of older women in Vietnam and Australia, the study found that older women in Vietnam had lower adjusted physical health than those in Australia highlighted a need for more interventions to promote their physical health. The deteriorated physical health of older women in Vietnam may be related to their experience of wars, lack of resources after wars, family care burdens—a primary responsibility of women in this country, chronic diseases [24] and violence experiences [25]. However, their adjusted mental health was similar. This finding is interesting when the dramatic life experiences such as wars, the difficulty of life after wars, and the recent rapid socio-economic changes experienced by older Vietnamese women is taken into consideration. Recent research has shown that exposure to life stressors, and those with adaptive coping strategies [26] and better levels of resilience [27] would be less likely to experience mental health problems. Coping strategies and resilience among older women in Vietnam might be potential explanations for this similarity. However, as the study used an interview-administered, self-reporting questionnaire to measure mental health, the participants in Vietnam may have under-reported. Therefore, future investigation into coping and resilience among older women in Vietnam is recommended.

Concerning factors associated with HRQOL, in line with current literature, the study delivered the same results for both populations: the number of chronic diseases [28] and diet [29] were associated with physical health; physical activity levels were linked with mental health [30]. This finding suggests that those with more chronic diseases, and a less healthy diet would have a lower level of physical health. Conversely, undertaking regular daily physical activity would had higher levels of mental health. This finding also suggested that interventions to manage chronic diseases, to promote healthy diet, and to moderate physical activity available in Australia can be considered to adopt and modify for Vietnam.

However, first, the relationships between physical activities, exercise, BMI and physical health were inconsistent among women in Vietnam and Australia. Findings from Australian dataset were in line with current literature, suggesting increased physical activity levels [30–32], frequent exercise [33], and normal BMIs [34–36] are linked to better physical health. However, data from Vietnam did not support this finding. To explain for this inconsistency, future study should explore how older women in Vietnam participate in physical activity and exercise. In addition, as a side note, because the number of obese women in Vietnam is small, the adverse influence of obesity on physical health could not be explored in this study.

A second difference among women in Vietnam and Australia is found in the relationship between age and physical health. As seen in Table 1, older women in Vietnam were less likely to practice exercise than those in Australia. Thus, it is expected that aging could reduce the physical health quicker in Vietnamese sample and so the association became significant in the data analysis. A third difference is found in the relationship between chronic diseases and mental health in the Vietnamese dataset—consistent with current literature [28]—but could not find it in Australian dataset. Fourth, while literature shows that age and alcohol drinking were linked to mental health [37], this association existed only in the Australian dataset. A possible explanation is that the association between age and mental health are inconsistent across the populations. In addition, the prevalence of women drinking alcohol was low in the Vietnamese dataset and the number of those suffering chronic disease among the Australian women was small: significant associations could thus not be found in this low extent. Finally, as personal characteristics, lifestyles, and chronic disease could explain variance of health among older women in Australia better than those in Vietnam (R^2^ = 32.1% vs. 16.9% for physical health and 21.4% vs. 10.1% for mental health), cultural factors may have contributed to the variation of HRQOL and so future research into cultural contribution in HRQOL is recommended.

### Limitations

It is always necessary to justify a study’s findings within its limitations. First, this study is cross-sectional and so does not allow for the confirmation of causal relationships like a prospective study design does. Second, as a self-reported questionnaire was used, social desired, reporting and recalling bias may occur. Finally, as data was not selected from a nationwide sample, generalisation of the study’s findings is limited to the study populations and the populations having similar characteristics to the study populations. However, the study also have several strengths. First, same measurements were used for both datasets and data were collected at an equivalent period. In addition, the study included socio-demographic characteristics, lifestyles and the number of chronic diseases, and applied advanced statistical approaches in data analysis to control for potential confounders and covariates. These steps improve accuracy of comparison findings [38]. As clinical implications, the study findings suggest that resources to improve health and wellbeing for older Australia can be adopted for use in Vietnam; future interventions in Vietnam should target healthy diet, moderate physical activity and be tailored for different age groups, marital statuses and the number of chronic diseases experienced for better HRQOL. These interventions will bring more benefits for elderly, married women with more chronic diseases. Findings from this study may also be beneficial for other populations, which have similar characteristics with the study populations.

## Abbreviations

HRQOL: health-related quality of life
SF-12: Short-Form 12
GLM: general linear models
BMI: body mass index
SPSS: statistical package for social sciences
